# The impact of simulation-based training in medical education: A review

**DOI:** 10.1097/MD.0000000000038813

**Published:** 2024-07-05

**Authors:** Chukwuka Elendu, Dependable C. Amaechi, Alexander U. Okatta, Emmanuel C. Amaechi, Tochi C. Elendu, Chiamaka P. Ezeh, Ijeoma D. Elendu

**Affiliations:** a Federal University Teaching Hospital, Owerri, Nigeria; b Igbinedion University, Okada, Nigeria; c Imo State University, Owerri, Nigeria; d Madonna University, Elele, Nigeria; e University of Port Harcourt, Choba, Nigeria.

**Keywords:** clinical skills, healthcare simulation, medical education, patient safety, simulation-based training

## Abstract

Simulation-based training (SBT) has emerged as a transformative approach in medical education, significantly enhancing healthcare professionals’ learning experience and clinical competency. This article explores the impact of SBT, tracing its historical development and examining the various types of simulations utilized today, including high-fidelity mannequins, virtual reality environments, standardized patients, and hybrid simulations. These methods offer a safe and controlled environment for students to practice and hone technical and non-technical skills, ultimately improving patient safety and clinical outcomes. The benefits of SBT are manifold, including enhanced skill acquisition, error reduction, and the opportunity for repeated practice without risk to actual patients. Immediate feedback and structured debriefing further solidify learning, making Simulation an invaluable tool in medical education. However, the implementation of SBT is challenging. It requires substantial financial investment, specialized equipment, and trained faculty. Additionally, there are concerns about the realism of simulations and the transferability of skills to real-world clinical settings. Despite these challenges, numerous case studies and empirical research underscore the effectiveness of SBT compared to traditional methods. Looking ahead, advancements in technology, such as artificial intelligence and improved virtual reality applications, promise to enhance the efficacy and accessibility of simulation training. The integration of Simulation with other training modalities and its adoption in diverse global contexts highlight its potential to revolutionize medical education worldwide. This article affirms the crucial role of SBT in preparing the next generation of healthcare professionals and its ongoing evolution driven by technological innovations.

## 1. Introduction and background

Simulation-based training (SBT) has become an integral component of medical education, transforming how healthcare professionals are trained by providing realistic, immersive learning experiences that closely mimic clinical scenarios. This approach allows learners to develop and refine both technical and non-technical skills in a safe and controlled environment, significantly enhancing their preparedness for real-life medical situations. Historically, medical education relied heavily on the apprenticeship model, where students learned through direct patient care under the supervision of experienced clinicians. This model has inherent limitations, including the variability of clinical experiences and the potential risk to patient safety.^[1]^ As medical knowledge expanded and healthcare became more complex, the need for standardized, reproducible training methods became evident.^[2]^ Using Simulation in medical training dates back to the 1960s with the development of Resusci Anne, a mannequin designed for practicing cardiopulmonary resuscitation (CPR).^[3]^ Since then, simulation technology has advanced dramatically, encompassing many modalities, including high-fidelity mannequins, virtual reality (VR) environments, standardized patients, and hybrid simulations. These tools allow learners to practice and master diverse clinical skills, from basic procedures to complex surgical techniques.^[4,5]^ High-fidelity simulators are sophisticated, lifelike mannequins capable of mimicking various physiological responses and medical conditions. These simulators allow learners to practice procedures such as intubation, chest tube insertion, and advanced cardiac life support in a realistic setting.^[6]^ Studies have shown that high-fidelity Simulation improves skill acquisition and retention compared to traditional training methods.^[7]^ Moreover, it enhances learners’ confidence and reduces anxiety, critical for effective performance in high-stakes clinical environments.^[8]^ VR and augmented reality (AR) technologies are also gaining traction in medical education. These tools create immersive, interactive environments where learners can practice surgical techniques, navigate complex anatomical structures, and manage critical care scenarios without the constraints of physical simulators.^[9,10]^ VR training has been particularly beneficial in surgical education, where it provides a risk-free platform for practicing intricate procedures and allows for repeated practice until proficiency is achieved.^[11]^ Research indicates that VR training can improve surgical performance and reduce the learning curve for complex procedures.^[12]^ Standardized patients, individuals trained to portray real patients consistently and accurately, are another valuable component of SBT. They allow learners to practice history taking, physical examination, and communication skills in a realistic, patient-centered context.^[13]^ Standardized patients have been shown to improve learners’ diagnostic accuracy, communication skills, and overall clinical competence.^[14,15]^ Furthermore, it allows for assessing learners in a controlled, standardized manner, providing valuable feedback, and identifying areas for improvement.^[16]^ Hybrid simulations combine various simulation modalities to create comprehensive, realistic training scenarios. For instance, a hybrid simulation might involve a standardized patient with a high-fidelity mannequin to simulate a patient undergoing a medical emergency.^[17]^ This approach allows learners to integrate technical and non-technical skills, such as teamwork and communication, in a realistic clinical context.^[18]^ Hybrid simulations have been shown to enhance learners’ ability to manage complex clinical situations and improve overall performance.^[19]^ The benefits of SBT extend beyond skill acquisition. One of the most significant advantages is the opportunity for deliberate practice, where learners can repeatedly perform tasks and refine their skills without risk to patients.^[20]^ This repetition is crucial for developing proficiency and ensuring that skills are retained over time.^[21]^ Additionally, Simulation provides a safe environment for learners to make and learn from mistakes, essential for effective learning.^[22]^ Immediate feedback and structured debriefing are key components of SBT. After each simulation session, learners participate in debriefing sessions, receiving feedback on their performance, reflecting on their actions, and discussing ways to improve.^[23]^ This process is critical for reinforcing learning, correcting errors, and promoting reflective practice.^[24]^ Studies have shown that debriefing significantly enhances learning outcomes and improves clinical performance.^[25]^ Despite its many advantages, SBT faces several challenges. One of the primary barriers is the high cost associated with purchasing and maintaining simulation equipment and the need for specialized facilities and trained personnel.^[26]^ This can be a significant financial burden for educational institutions, particularly those in resource-limited settings.^[27]^ Furthermore, while advanced, the realism of simulations still cannot fully replicate the complexities and unpredictability of real-life clinical situations.^[28]^ This raises concerns about transferring skills acquired through Simulation to actual patient care.^[29]^ Another challenge is the need for faculty training and development. Effective SBT requires instructors to be proficient in the technical aspects of Simulation and skilled in facilitating debriefing and providing constructive feedback.^[30]^ This necessitates ongoing professional development and support for educators, which can be resource-intensive.^[31]^ Despite these challenges, the evidence supporting the effectiveness of SBT is compelling. Numerous studies have demonstrated that Simulation improves clinical skills, enhances patient safety, and leads to better clinical outcomes compared to traditional training methods.^[32–34]^ For example, a meta-analysis of simulation-based education in medical schools found that students trained in Simulation performed significantly better in technical and non-technical skills assessments than those who received traditional training.^[35]^ Looking ahead, technological advancements promise further to enhance the capabilities and accessibility of SBT. Artificial intelligence (AI) is poised to play a significant role in the future of Simulation, offering opportunities for personalized learning experiences and adaptive training programs that cater to individual learners’ needs.^[36]^ AI-driven simulations can provide real-time feedback, monitor learners’ progress, and adjust the difficulty of scenarios based on performance, creating a more tailored and effective training experience.^[37]^ Improved VR and AR applications are also on the horizon, offering even more realistic and immersive training environments.^[38]^ These technologies can potentially revolutionize medical education by giving learners unprecedented access to complex clinical scenarios and rare medical conditions.^[39]^ Integrating Simulation with other training modalities, such as problem-based learning and interdisciplinary training programs, can create a more holistic and comprehensive educational experience.^[40]^ The global adoption of SBT is also a key area of focus. While high-income countries have widely embraced Simulation, its adoption in low- and middle-income countries has been slower due to financial and infrastructural constraints.^[41]^ Efforts to increase access to simulation training in these regions are crucial for improving healthcare education and outcomes globally.^[42]^ Cross-cultural adaptations of simulation scenarios and the development of cost-effective simulation solutions can help bridge this gap and ensure that all healthcare professionals have access to high-quality training.^[43]^

## 2. Statement of concrete aims

1. Provide an overview of the historical development of Simulation in medical education, highlighting key milestones and advancements.
2. Explore the various simulation modalities used in medical training, including high-fidelity simulators, VR environments, standardized patients, and hybrid simulations.
3. Discuss the benefits of SBT, including enhanced skill acquisition, improved patient safety, and creating a safe learning environment.
4. Identify and analyze the challenges and limitations of SBT, such as cost, realism, and faculty training requirements.
5. Present case studies and empirical research findings that illustrate the effectiveness of SBT in improving clinical competency and patient outcomes.
6. Discuss future directions and innovations in SBT, including AI integration, VR technology advancements, and efforts to increase global access to simulation training.

## 3. Materials and methods

### 3.1. Literature review

A comprehensive literature review was conducted to gather relevant studies, articles, and research findings related to the impact of SBT in medical education. The search was conducted across electronic databases, including PubMed, Google Scholar, and Web of Science, using keywords such as “simulation-based training,” “medical education,” “simulation in healthcare,” and “virtual reality simulation.”

The inclusion criteria for selecting studies were:

- Articles published in peer-reviewed journals.
- Studies focusing on SBT methods in medical education.
- Research articles, case studies, systematic reviews, and meta-analyses.
- Studies published in English.

Exclusion criteria included:

- Articles not relevant to medical education or simulation training.
- Non-peer-reviewed literature, such as conference abstracts and editorials.
- Studies with insufficient data or unclear methodology.

The search was conducted from the inception of relevant databases to the present date, with no restrictions on publication year. The initial search yielded many articles, screened based on titles and abstracts for relevance to the topic. Full-text articles meeting the inclusion criteria were then reviewed in detail to extract relevant information, including study objectives, methodology, findings, and conclusions. Data from selected studies were synthesized and analyzed to identify common themes, trends, and key findings related to the impact of SBT in medical education. This process involved categorizing studies based on simulation modalities, learning outcomes, and study populations. Furthermore, references cited in selected articles were reviewed to identify additional relevant studies that may have yet to be captured in the initial search. This iterative process ensured a comprehensive review of the existing literature on SBT in medical education.

## 4. Historical background of SBT

Medical education has undergone significant transformations over the centuries, from rudimentary apprenticeships to highly structured and technologically advanced training programs. This evolution reflects broader changes in society, technology, and our understanding of health and disease. One of the most profound changes in recent decades has been the introduction and widespread adoption of SBT. This innovative approach has revolutionized how healthcare professionals are educated, enhancing clinical skills, improving patient safety, and addressing the limitations of traditional training methods. Training was largely an informal process in the earliest days of medical practice. Aspiring physicians learned through apprenticeships, working alongside experienced practitioners. This hands-on approach provided practical experience but lacked standardization and formal assessment. The knowledge base of medicine was limited, and much of the training was observational, with little emphasis on scientific principles. Medical texts such as those by Hippocrates and Galen were influential, but their teachings were often interpreted and applied inconsistently.^[1]^ The Renaissance period marked a significant turning point in medical education. The rediscovery of classical texts and the rise of humanism emphasized the importance of empirical observation and critical thinking. Medical schools began to emerge, particularly in Europe, providing more formalized education. Anatomical dissections became a core training component, greatly enhancing the understanding of human anatomy and physiology. Figures such as Andreas Vesalius were instrumental in promoting a more scientific approach to medicine, challenging long-held beliefs, and advocating for direct observation and experimentation.^[2]^ The 19th century saw further advancements with the establishment of modern medical schools and the introduction of rigorous curricula. The publication of the Flexner Report in 1910 was a watershed moment. Commissioned by the Carnegie Foundation, Abraham Flexner’s report criticized the inconsistent standards of medical education in the United States and Canada. It called for substantial reforms, including higher admission standards, a more scientific medical education approach, and laboratory and clinical training integration. The report led to the closure of many substandard medical schools and establishing stricter accreditation standards, laying the groundwork for modern medical education.^[3]^ By the mid-20th century, medical education had become more standardized and scientifically grounded. The curriculum was heavily based on biomedical sciences, with students spending the first 2 years in classroom and laboratory settings, followed by clinical rotations in various specialties. While effective in imparting theoretical knowledge, this model had limitations in providing practical skills and real-life clinical experience. Training often occurred on actual patients, raising ethical concerns and potential risks to patient safety. These challenges highlighted the need for innovative training methods to provide practical experience without compromising patient care.^[4]^ The introduction of Simulation in medical education addressed many of these challenges. The roots of Simulation can be traced back to the mid-20th century with the development of simple training aids such as Resusci Anne, a mannequin designed for practicing CPR. Developed in the 1960s by Asmund Laerdal in collaboration with Dr Peter Safar, Resusci Anne provided a realistic, hands-on tool for learning life-saving techniques. This innovation demonstrated the potential of Simulation to enhance practical skills training in a safe and controlled environment.^[5]^ The 1970s saw further advancements with the creation of more sophisticated simulators. Harvey, a cardiology patient simulator developed at the University of Miami, was designed to teach medical students about heart disease and cardiac examination. Harvey could replicate various heart sounds and conditions, allowing students to practice diagnostic skills on a realistic model. This period also saw the development of anesthesia simulators, which provided a safe environment for practicing anesthesia techniques and managing complications.^[6]^ A significant leap forward occurred in the 1990s with the advent of high-fidelity patient simulators. These advanced mannequins could mimic a wide range of physiological responses and medical conditions, from breathing and heart rhythms to trauma and drug interactions. High-fidelity simulators such as SimMan, introduced by Laerdal, provided unprecedented realism in medical training. They allowed learners to engage in complex clinical scenarios, enhancing technical and decision-making skills. Studies began to show that training with high-fidelity simulators improved skill acquisition, retention, and learner confidence compared to traditional methods.^[7]^ The late 20th and early 21st centuries saw the integration of VR and AR technologies into medical training. VR and AR offered immersive environments for practicing surgical techniques, navigating anatomical structures, and managing critical care scenarios. These technologies provided a risk-free platform for repeated practice, allowing learners to refine their skills until proficiency. Research indicated that VR training could significantly improve surgical performance and reduce the learning curve for complex procedures.^[8]^ Using standardized patients, actors trained to portray real patients consistently became another integral component of SBT. Standardized patients provided realistic scenarios for practicing history taking, physical examination, and communication skills. This method emphasized the importance of patient-centered care and allowed for the assessment and improvement of both technical and non-technical skills. Studies have shown that training with standardized patients enhances diagnostic accuracy, communication skills, and overall clinical competence.^[9]^ Hybrid simulations, which combine various simulation modalities, emerged as a comprehensive approach to medical training. For example, a hybrid simulation might involve a standardized patient interacting with a high-fidelity mannequin to simulate a patient experiencing a medical emergency. This approach allows learners to integrate technical and critical non-technical skills, such as teamwork and communication, in a realistic clinical context. Hybrid simulations have been shown to enhance learners’ ability to manage complex clinical situations and improve overall performance.^[10]^ The benefits of SBT are numerous and well-documented. One of the most significant advantages is the ability to provide a safe and controlled environment for learning. In traditional clinical training, students often learn about real patients, which carries inherent risks and limitations. Simulation eliminates these risks, allowing learners to practice and refine their skills without endangering patient safety. This is particularly important for high-stakes procedures and emergencies where mistakes can have serious consequences.^[11]^ SBT also supports deliberate practice, where learners can repeatedly perform tasks and receive immediate feedback. This repetition is crucial for developing proficiency and ensuring that skills are retained over time. Structured debriefing sessions following simulations provide opportunities for reflection, discussion, and learning from mistakes, further reinforcing learning outcomes. Research has consistently shown that SBT improves clinical skills, enhances patient safety, and improves clinical outcomes than traditional training methods.^[12]^ Despite its many benefits, implementing SBT is not without challenges. One of the primary barriers is the high cost associated with purchasing and maintaining simulation equipment and facilities. This can be a significant financial burden for educational institutions, particularly those in resource-limited settings. Additionally, while advanced, the realism of simulations still cannot fully replicate the complexities and unpredictability of real-life clinical situations. This raises concerns about transferring skills acquired through Simulation to actual patient care.^[13]^ Another challenge is the need for faculty training and development. Effective SBT requires instructors proficient in Simulation’s technical aspects and skilled in facilitating debriefing and providing constructive feedback. This necessitates ongoing professional development and support for educators, which can be resource-intensive. Integrating Simulation into existing curricula requires careful planning and coordination to ensure it complements traditional training methods and enhances overall learning outcomes.^[14]^ Looking to the future, technological advancements promise further to enhance the capabilities and accessibility of SBT. AI is poised to play a significant role, offering opportunities for personalized learning experiences and adaptive training programs that cater to individual learners’ needs. AI-driven simulations can provide real-time feedback, monitor learners’ progress, and adjust the difficulty of scenarios based on performance, creating a more tailored and effective training experience.^[15]^ Improved VR and AR applications are also on the horizon, offering even more realistic and immersive training environments. These technologies can potentially revolutionize medical education by giving learners unprecedented access to complex clinical scenarios and rare medical conditions.^[16]^ Global adoption of SBT is another crucial area of focus. While high-income countries have widely embraced Simulation, its adoption in low- and middle-income countries has been slower due to financial and infrastructural constraints. Efforts to increase access to simulation training in these regions are essential for improving healthcare education and outcomes globally. Cross-cultural adaptations of simulation scenarios and the development of cost-effective simulation solutions can help bridge this gap and ensure that all healthcare professionals have access to high-quality training.^[17]^

## 5. Types of simulation in medical education

High-fidelity simulations are among the most advanced forms of Simulation in medical education. These simulations use mannequins and advanced patient simulators to replicate various physiological responses and medical conditions. High-fidelity mannequins, such as SimMan by Laerdal, can simulate breathing, heart rhythms, and even complex medical emergencies like cardiac arrest or respiratory failure. These simulators have sophisticated software that allows instructors to control and monitor various parameters, providing realistic and dynamic training scenarios. High-fidelity simulations offer several key advantages. First, they provide a safe environment for learners to practice and refine their skills without risking patient safety. Students can repeatedly perform procedures such as intubation, intravenous insertion, and defibrillation until they achieve proficiency. This repetitive practice is crucial for building muscle memory and ensuring that skills are retained over time. High-fidelity simulations also allow replicating rare and complex medical conditions that students might not encounter during their clinical rotations. This exposure is invaluable for preparing learners to handle various clinical situations.^[1]^ Additionally, high-fidelity simulations enhance decision-making and critical thinking skills. During these simulations, learners must assess the patient’s condition, make diagnostic decisions, and implement real-time treatment plans. This process mirrors the demands of real-life clinical practice, where rapid and accurate decision-making is essential. Studies have shown that training with high-fidelity simulators improves clinical performance, increases learner confidence, and reduces errors in actual patient care.^[2]^ VR simulations represent another innovative approach in medical education. VR simulations use computer-generated environments to create immersive training scenarios. Learners can navigate through virtual hospitals, interact with virtual patients, and perform various medical procedures. VR technology provides a highly engaging and interactive learning experience, which has been shown to enhance motivation and knowledge retention. One of the significant benefits of VR simulations is their ability to provide consistent and standardized training experiences. Unlike real-life clinical settings, where variability in patient conditions and instructor methods can affect learning outcomes, VR simulations offer uniform scenarios that ensure all learners receive the same training. This standardization is particularly beneficial for assessing and comparing learner performance.^[3]^ VR simulations also offer unparalleled flexibility. Training scenarios can be easily modified to suit different learning objectives, skill levels, and specialties. For example, surgical trainees can practice laparoscopic or robotic surgery in a virtual environment, gaining valuable experience without the risks associated with live surgery. VR simulations can also incorporate gamification elements, such as scoring systems and feedback, to enhance learner engagement and motivation.^[4]^ Furthermore, VR technology is increasingly being used to simulate interprofessional training scenarios. These simulations bring together medical students, nurses, pharmacists, and other healthcare professionals to practice teamwork and communication in a virtual setting. Interprofessional education is crucial for promoting collaboration and improving patient care, and VR provides a unique platform for these training experiences.^[5]^ Standardized patients are another critical component of SBT. Standardized patients are actors trained to portray real patients with specific medical conditions and histories consistently. They provide realistic scenarios for learners to practice history taking, physical examination, and communication skills. Standardized patients are used extensively in undergraduate and postgraduate medical education, licensure, and certification exams. The use of standardized patients offers several advantages. First, it provides a highly realistic and interactive learning experience. Learners can practice interpersonal skills in a controlled environment, such as empathy, active listening, and patient education. This is particularly important for developing patient-centered care skills and building patient trust and rapport.^[6]^ Standardized patients also provide valuable feedback to learners. After each encounter, actors can offer insights into the learner’s communication style, clinical approach, and overall performance. This feedback is crucial for identifying areas for improvement and enhancing clinical competence. Studies have shown that training with standardized patients improves diagnostic accuracy, patient interaction skills, and overall clinical performance.^[7]^ Additionally, standardized patients are used for formative and summative assessments. Objective Structured Clinical Examinations (OSCEs) are a common assessment method that uses standardized patients to evaluate clinical skills in a structured and standardized manner. OSCEs comprehensively assess a learner’s ability to perform clinical tasks, make diagnostic decisions, and communicate effectively with patients.^[8]^ Hybrid simulations combine various simulation modalities to create comprehensive and realistic training scenarios. For example, a hybrid simulation might involve a standardized patient interacting with a high-fidelity mannequin to simulate a patient experiencing a medical emergency. This approach allows learners to integrate technical and critical non-technical skills, such as teamwork and communication, in a realistic clinical context. Hybrid simulations offer several key benefits. First, they provide a holistic training experience that mirrors the complexities of real-life clinical practice. Learners must perform technical procedures, communicate with patients and team members, and make clinical decisions under pressure. This integrated approach enhances overall clinical competence and prepares learners for the multifaceted nature of patient care.^[9]^ Hybrid simulations also promote interprofessional education and collaboration. By involving multiple healthcare professionals, these simulations provide opportunities for learners to practice teamwork and communication in a realistic clinical environment. Effective interprofessional collaboration is essential for improving patient outcomes, and hybrid simulations provide a valuable platform for developing these skills.^[10]^ Moreover, hybrid simulations can be tailored to specific learning objectives and specialties. For example, a hybrid simulation for emergency medicine might involve a standardized patient presenting with chest pain, followed by the need for rapid assessment and intervention using a high-fidelity mannequin. This scenario would allow learners to practice various skills, from history-taking and physical examination to critical decision-making and procedural intervention.^[11]^ The implementation of SBT in medical education has been supported by a growing body of research demonstrating its effectiveness. Numerous studies have shown that SBT improves clinical skills, enhances patient safety, and leads to better clinical outcomes than traditional training methods. Meta-analyses and systematic reviews have consistently shown that simulation-based education with deliberate practice yields superior results in skill acquisition and retention.^[12]^ Despite the clear benefits, the widespread adoption of SBT faces several challenges. One of the primary barriers is the high cost associated with purchasing and maintaining simulation equipment and facilities. High-fidelity mannequins, VR systems, and standardized patient programs require significant financial investment, which can burden educational institutions, particularly in resource-limited settings.^[13]^ Another challenge is the need for faculty training and development. Effective SBT requires instructors proficient in Simulation’s technical aspects and skilled in facilitating debriefing and providing constructive feedback. This necessitates ongoing professional development and support for educators, which can be resource-intensive. Additionally, integrating Simulation into existing curricula requires careful planning and coordination to ensure it complements traditional training methods and enhances overall learning outcomes.^[14]^ Technological advancements continue to shape the future of SBT. AI is expected to play a significant role, offering opportunities for personalized learning experiences and adaptive training programs. AI-driven simulations can provide real-time feedback, monitor learners’ progress, and adjust the difficulty of scenarios based on performance, creating a more tailored and effective training experience.^[15]^ Improved VR and AR applications also promise to enhance the realism and immersion of simulation environments, providing learners with access to complex clinical scenarios and rare medical conditions.^[16]^ Efforts to increase access to simulation training globally are also essential. While high-income countries have widely embraced Simulation, its adoption in low- and middle-income countries has been slower due to financial and infrastructural constraints. Initiatives to develop cost-effective simulation solutions and provide training resources in these regions are crucial for improving healthcare education and outcomes worldwide.^[17]^ Table 1 illustrates current SBT modalities, including their geographical spread, benefits, and limitations, to aid in understanding the landscape of SBT in healthcare education and practice.

**Table 1:** Current SBT modalities, their geographical spread, benefits, and individual limitations.

SBT modality	Description	Geographical spread	Benefits	Limitations
High-fidelity	Utilizes advanced patient simulators that closely mimic human physiology and responses	Widely adopted in North America, Europe, and Asia	- Realistic patient scenarios - Immersive learning experience - Opportunities for hands-on practice with medical equipment - Real-time feedback and assessment	- Cost-prohibitive for many institutions - Requires specialized facilities and equipment - May lack scalability for large-scale training programs - Maintenance and repair of simulators can be challenging
VR	Creates computer-generated environments and scenarios that users can interact with using VR headsets	Increasing adoption worldwide, particularly in developed regions	- Immersive, lifelike simulations - Safe and controlled learning environment - Accessible anytime, anywhere - Opportunity for repeated practice - Real-time feedback and assessment	- Initial setup costs can be high - Requires technical expertise to develop and maintain simulations - Potential for simulator sickness or discomfort - Limited tactile feedback compared to real-world interactions
AR	Overlays digital information onto the real-world environment, enhancing learners’ situational awareness	Growing adoption in healthcare education and training	- Enhances realism by integrating digital content with the physical environment - Facilitates hands-on learning experiences - Improves spatial awareness and decision-making skills	- Limited availability of AR hardware and software - Requires specialized training for users - May be prone to technical glitches or malfunctions - Limited depth perception and immersion compared to VR
Standardized patients	Actors trained to portray patients with specific medical conditions and histories	Commonly used in North America and Europe, expanding globally	- Provides realistic interactions with patients - Allows for the practice of communication and interpersonal skills - Opportunities for empathy and cultural sensitivity training - Flexibility in scenario design and customization	- Cost and resource-intensive to train standardized patients - Variation in performance quality among standardized patients - Limited scalability for large-scale training programs - May not fully replicate the complexity of real patient encounters
Hybrid simulations	Combines multiple simulation modalities, such as high-fidelity mannequins, with virtual or augmented reality	Emerging trend with increasing adoption in various regions	- Offers diverse learning experiences - Integrates strengths of different modalities - Provides comprehensive training across multiple skill domains - Enhances realism and immersion	- Complexity of setup and coordination among different modalities - Potential for technical integration challenges - Requires specialized expertise to design and facilitate hybrid simulations - Cost and resource-intensive

This comprehensive table overviews various SBT modalities, their geographical spread, benefits, and limitations. It underscores the diverse range of options available to educators and healthcare professionals. Importantly, it highlights the need to consider cost, realism, and scalability factors when selecting appropriate modalities for training programs. By considering these factors, educators and healthcare professionals can make informed decisions that best meet their training needs.

AR = augmented reality, SBT = simulation-based training, VR = virtual reality.

## 6. Benefits of SBT

One of the primary benefits of SBT is enhanced skill acquisition. This encompasses technical skills, such as surgical procedures and clinical interventions, and non-technical skills, including communication, teamwork, and decision-making. Technical skills are fundamental to medical practice, and SBT provides an ideal platform for their development. High-fidelity simulators, VR environments, and standardized patients allow learners to practice various procedures in a controlled setting. For instance, medical students can perform intubations, catheterizations, and suturing on mannequins that replicate human anatomy and physiological responses. This hands-on practice is crucial for building proficiency and confidence. Studies have shown that repeated practice with simulators leads to improved performance and higher skill retention compared to traditional methods.^[1]^ Non-technical skills are equally important in healthcare, where effective communication, teamwork, and decision-making can significantly impact patient outcomes. SBT provides scenarios that mimic real-life clinical settings, allowing learners to practice these skills in context. For example, team-based simulations require participants to collaborate, delegate tasks, and communicate effectively under pressure. These scenarios help develop crucial interpersonal skills essential for functioning in multidisciplinary healthcare teams. Research indicates simulation training improves teamwork, enhances communication skills, and fosters better clinical decision-making.^[2]^ Increased patient safety is another critical benefit of SBT. By allowing learners to practice and refine their skills in a risk-free environment, Simulation helps reduce the incidence of errors in actual clinical practice. One significant aspect of this is error reduction. In traditional training models, students often learn by performing procedures on real patients, which can lead to mistakes that compromise patient safety. Simulation eliminates this risk by providing a safe space for learners to make and learn from their mistakes without causing harm.^[3]^ SBT also contributes to improved clinical outcomes. Well-trained healthcare professionals are less likely to make errors and more likely to provide high-quality care. Studies have demonstrated that simulation training leads to better clinical performance, including more accurate diagnoses, timely interventions, and effective management of medical emergencies. For instance, a study on the impact of simulation training on neonatal resuscitation found that trainees who practiced with high-fidelity simulators performed significantly better than those who did not, resulting in improved neonatal outcomes.^[4]^ A safe learning environment is another major advantage of SBT. This environment allows learners to practice repeatedly and learn from their mistakes without the pressure of real-life consequences. Repeated practice is essential for mastering complex medical procedures and building the confidence to perform them accurately in clinical settings. High-fidelity simulators and VR environments can be used multiple times, allowing learners to practice until they achieve proficiency.^[5]^ Moreover, the ability to learn from mistakes is a crucial aspect of SBT. In traditional clinical training, mistakes can seriously affect patients, creating a high-stress learning environment. Simulation mitigates this issue by providing a safe space where errors can be made and corrected without risk to patient safety. This fosters a more positive learning experience and encourages learners to take the necessary risks to improve their skills. Research shows that the opportunity to make and learn from mistakes in a simulated environment enhances skill acquisition and retention.^[6]^ Immediate feedback and assessment are integral components of SBT. Real-time performance evaluation allows learners to receive instant feedback on their actions, facilitating immediate correction and improvement. This is particularly beneficial in high-stakes scenarios where quick decision-making and precise execution are critical. For example, during a simulated cardiac arrest, learners can receive immediate feedback on the quality of chest compressions, ventilation, and drug administration, enabling them to adjust their techniques on the spot.^[7]^ Structured debriefing sessions following simulations are another critical aspect of immediate feedback and assessment. Debriefing involves a guided discussion where learners reflect on their performance, identify areas for improvement, and receive constructive instructor feedback. This process helps consolidate learning, reinforce best practices, and address gaps in knowledge or skills. Studies have shown that debriefing is one of the most effective methods for enhancing learning outcomes in SBT.^[8]^ A growing body of evidence supports the benefits of SBT. Numerous studies and meta-analyses have demonstrated its effectiveness in improving both technical and non-technical skills, enhancing patient safety, and providing a positive learning environment. For instance, a systematic review of simulation-based medical education found that it significantly improves clinical skills, increases learner confidence, and reduces the incidence of errors in clinical practice.^[9]^

## 7. Challenges and limitations of SBT

One of the most prominent challenges is the high cost and resource-intensive nature of SBT. Implementing and maintaining high-fidelity simulation programs requires substantial financial investment. Purchasing sophisticated simulators, such as high-fidelity mannequins and VR systems, can be prohibitively expensive for many educational institutions, especially those with limited budgets. For instance, a single high-fidelity mannequin can cost upwards of $50,000, not including the additional expenses for software, maintenance, and upgrades.^[1]^ Moreover, establishing dedicated simulation centers with advanced technology and facilities further adds to the financial burden. These centers require significant capital for construction, outfitting, and ongoing operational costs. Beyond the initial financial outlay, SBT requires specialized equipment and facilities that are often complex to manage and maintain. High-fidelity simulators need regular maintenance and updates to ensure they function correctly and provide accurate, realistic experiences. The technological infrastructure required for VR simulations also demands significant investment in hardware, software, and technical support to troubleshoot issues and maintain system functionality.^[2]^ These requirements can strain the resources of educational institutions, particularly those in resource-limited settings, limiting the widespread adoption of SBT. Another significant challenge is the need for faculty training. Effective SBT hinges on the proficiency of instructors in both the technical aspects of Simulation and the pedagogical skills necessary to facilitate learning. Instructors must be adept at operating complex simulation equipment, designing realistic training scenarios, and conducting structured debriefing sessions that provide valuable feedback. This requires comprehensive training programs for faculty, which can be time-consuming and resource-intensive.^[3]^ Institutions must invest in continuous professional development to ensure faculty remain up-to-date with the latest simulation technologies and educational methodologies. Integrating Simulation into existing curricula also presents a challenge. Developing a simulation-based curriculum that complements traditional teaching methods and enhances overall learning outcomes requires careful planning and coordination. Faculty must be involved in curriculum development to ensure that simulation activities align with learning objectives and provide meaningful educational experiences. This process often involves significant changes to the traditional educational structure, which can be resisted by faculty and administrators accustomed to conventional teaching methods.^[4]^ Another limitation of SBT is the potential gap in realism compared to clinical settings. While high-fidelity simulators and VR environments strive to replicate real-life scenarios as closely as possible, they can never fully capture the complexity and unpredictability of actual clinical practice. Simulated environments may lack certain elements in real clinical settings, such as the emotional stress of dealing with real patients, the variability of patient responses, and the intricacies of working within a live healthcare team.^[5]^ This limitation can lead to potential gaps in training, where learners might not experience all the nuances and challenges they would encounter in a real clinical setting. The transferability of skills learned in Simulation to real-life situations is another concern. While SBT has improved clinical skills and decision-making, there is still ongoing debate about how these skills transfer to actual patient care. Some studies suggest that the controlled and predictable nature of simulation scenarios may not fully prepare learners for the dynamic and often chaotic environment of a real clinical setting.^[6]^ For example, managing a simulated cardiac arrest scenario might not encompass the full spectrum of challenges presented by a real-life cardiac arrest, where unforeseen complications and human factors come into play. Despite these challenges and limitations, it is important to recognize the significant progress and improvements made possible by SBT. Institutions can mitigate some of these challenges through strategic planning and resource allocation. For instance, developing partnerships with technology companies, securing grants, and investing in cost-effective simulation solutions can help alleviate the financial burden. Additionally, incorporating blended learning approaches that combine Simulation with traditional clinical experiences can enhance the realism and transferability of skills.^[7]^ Furthermore, continuous evaluation and research into SBT are essential to address its limitations and optimize its effectiveness. By identifying and addressing gaps in training, educational institutions can refine their simulation programs to prepare learners for real-life clinical practice better. This includes ongoing assessment of the impact of simulation training on clinical performance and patient outcomes, as well as exploring innovative solutions to enhance the realism and immersive quality of simulation experiences.^[8]^

## 8. Assessment and Evaluation of SBT

SBT in medical education has become essential for developing clinical skills and improving patient outcomes. As SBT continues to gain traction, robust assessment and evaluation methods are critical to measure its effectiveness. Effective assessment and evaluation ensure the training programs’ quality and provide insights into areas for improvement and future development. The assessment of SBT typically involves evaluating both technical and non-technical skills. Technical skills include procedural competencies, such as performing surgical techniques or managing airway emergencies, while non-technical skills encompass communication, teamwork, and decision-making abilities. A multi-dimensional approach to assessment is essential to capture the complexity and breadth of competencies developed through SBT.^[1]^ One widely used method for assessing technical skills in SBT is the OSCE. OSCEs involve a series of stations where learners perform specific tasks or procedures under observation. These tasks are standardized and scored using checklists or rating scales, ensuring objective performance measurement. Studies have shown that OSCEs effectively evaluate clinical competencies acquired through simulation training and provide reliable and valid assessments.^[2]^ For example, a study by Higham^[3]^ demonstrated that OSCEs could effectively assess the skills of medical students and residents in simulated environments, leading to improved performance in real clinical settings. Another key method for evaluating SBT is direct observation with feedback. Instructors observe learners during simulation exercises and provide immediate, constructive feedback. This real-time assessment allows for identifying strengths and areas for improvement, facilitating a continuous learning process. Feedback is often supplemented by video recordings of the simulations, which learners can review to self-assess their performance. This method has enhanced learning outcomes and boosted learners’ confidence and competence.^[4]^ SBT also emphasizes assessing non-technical skills, such as communication and teamwork. The TeamSTEPPS (Team Strategies and Tools to Enhance Performance and Patient Safety) program is an example of a structured approach to training and assessing teamwork skills in healthcare settings. TeamSTEPPS utilizes simulation scenarios to train healthcare teams in effective communication, leadership, situational awareness, and mutual support. The program’s assessment tools, including the Team Performance Observation Tool, enable objective evaluation of team dynamics and performance during simulations.^[5]^ To evaluate the overall effectiveness of SBT programs, educators often use Kirkpatrick’s Four-Level Training Evaluation Model. This model assesses training programs at four levels: reaction, learning, behavior, and results. At the reaction level, learners’ satisfaction and engagement with the training are measured through surveys and feedback forms. The learning level assesses knowledge and skills acquisition through pre-and post-tests. The behavior level evaluates the application of learned skills in real clinical settings, often through follow-up assessments and observations. Finally, the results level measures the impact of the training on patient outcomes and organizational performance.^[6]^ Following simulation training, studies applying Kirkpatrick’s model have significantly improved learners’ skills and patient care quality.^[7]^ Evaluating the long-term impact of SBT on clinical practice and patient outcomes is crucial. Longitudinal studies tracking the performance of healthcare professionals over time can provide valuable insights into the sustained benefits of simulation training. For instance, a study by McGaghie et al tracked medical residents trained in CPR using high-fidelity simulators. The results showed that simulation-trained residents maintained their skills and performed better in real-life resuscitation scenarios even 1 year after the training.^[44]^ Such findings highlight the lasting impact of SBT on clinical competence. Despite the numerous benefits, assessing and evaluating SBT also present challenges. One major challenge is ensuring the validity and reliability of assessment tools. Standardized assessment instruments, such as checklists and rating scales, must be rigorously developed and validated to measure the intended competencies accurately. Additionally, inter-rater reliability must be established to ensure consistency in scoring across different evaluators.^[9]^ Another challenge is the potential for assessment bias. Instructors’ familiarity with learners or preconceived notions about their abilities can influence the objectivity of assessments. To mitigate this, blind assessments can be implemented, where the evaluator is unaware of the learner’s identity. Furthermore, using multiple evaluators and triangulating assessment data from various sources can enhance the objectivity and reliability of evaluations.^[10]^ Resource constraints also pose a challenge to the comprehensive assessment of SBT. High-fidelity simulations and OSCEs require significant time, equipment, and personnel investment. Developing and maintaining simulation centers, training standardized patients, and ensuring the availability of skilled instructors can be resource-intensive. However, the long-term benefits of improved clinical skills and patient safety often justify these investments.^[11]^

## 9. Ethical considerations in SBT

SBT in medical education offers significant benefits for developing clinical skills and enhancing patient safety. However, it also raises various ethical considerations that must be addressed to ensure the training process’s integrity, fairness, and effectiveness. Ethical concerns in SBT revolve around simulation technology, the treatment of learners and standardized patients, the design and implementation of simulation scenarios, and the overall impact on patient care. One primary ethical consideration in SBT is informed consent. Just as patients must provide informed consent for medical procedures, learners and standardized patients involved in simulation exercises should be fully informed about the nature of the simulations, potential risks, and the training objectives. This includes informing participants about the use of video recordings, the scope of their involvement, and any assessment methods employed. Ensuring informed consent respects the participants’ autonomy and upholds ethical standards in educational practice.^[1]^ Confidentiality is another critical ethical issue. The data generated during simulations, including performance evaluations and video recordings, must be handled with the same level of confidentiality as patient medical records. This data should be securely stored and only accessible to authorized personnel. Moreover, any use of simulation data for research purposes should be de-identified to protect participants’ privacy. Maintaining confidentiality fosters a safe learning environment where participants feel comfortable engaging fully in the training process without fear of undue exposure or judgment.^[2]^ The ethical treatment of standardized patients is also a key concern. Standardized patients, often laypersons trained to simulate real patient cases, play a crucial role in medical education. They must be treated respectfully and provided with adequate training and support to ensure their well-being. This includes preparing them for their roles’ emotional and psychological demands and offering debriefing sessions to address any distress that may arise from their participation. Additionally, standardized patients should be fairly compensated for their time and effort.^[3]^ The design and implementation of simulation scenarios must be ethically sound to avoid causing undue stress or harm to learners. Scenarios should be realistic and relevant but also designed to ensure psychological safety. This involves creating a supportive environment where learners can make mistakes and learn from them without fear of humiliation or punitive consequences. Educators must balance the need for challenging simulations with the responsibility to avoid overwhelming or traumatizing participants. Providing thorough debriefing sessions where learners can reflect on their experiences and receive constructive feedback is essential for their emotional and educational well-being.^[4]^ Bias and fairness in assessment are significant ethical issues in SBT. Assessment tools and evaluators must be free from biases that could unfairly influence learners’ evaluations. This includes addressing potential gender, racial, and cultural biases in both the design of simulation scenarios and the assessment process. Developing standardized assessment criteria and providing training for evaluators can help mitigate these biases. Ensuring fairness in assessment is critical for maintaining the credibility and ethical integrity of SBT programs.^[5]^ The impact of SBT on actual patient care raises additional ethical considerations. While simulation training is designed to improve clinical skills and enhance patient safety, there is an ethical obligation to ensure that the skills and knowledge gained through Simulation translate effectively into real-world practice. This involves continuously evaluating the effectiveness of SBT programs and making necessary adjustments to improve training outcomes. Moreover, educators must be vigilant in preventing a false sense of competence among learners, ensuring that they recognize the limitations of Simulation and understand the need for ongoing learning and supervision in clinical settings.^[6]^ Resource allocation is another ethical issue related to SBT. High-fidelity simulations and advanced technologies require significant financial and logistical resources. An ethical responsibility is ensuring these resources are used efficiently and equitably. This includes providing access to SBT for all learners, regardless of their institution’s financial status, and avoiding disparities in training opportunities. Collaboration and resource-sharing among institutions can help address these challenges and promote equitable access to high-quality simulation training.^[7]^ Finally, using Simulation for high-stakes assessments, such as certification or licensure exams, presents ethical challenges. High-stakes assessments can significantly impact learners’ careers, and it is crucial to ensure that these assessments are valid, reliable, and fair. Using standardized and transparent evaluation criteria and rigorous validation processes for assessment tools is essential to uphold the ethical standards of high-stakes testing.^[8]^

## 10. Patient perspectives on SBT

Patients generally express strong support for SBT, primarily because they believe it enhances the competence and preparedness of healthcare providers. When patients are aware that their doctors and nurses have undergone rigorous simulation training, they often feel more confident in the care they receive. This confidence stems from the understanding that SBT allows healthcare professionals to practice and perfect their skills without putting patients at risk. According to a study by Bradley,^[1]^ patients perceive healthcare providers trained with Simulation as more adept at handling complex and critical situations, which translates into higher levels of trust and reassurance. Moreover, patient safety is a significant concern for many patients, and SBT is viewed as a valuable tool in mitigating medical errors. Simulation provides a platform for healthcare workers to experience and learn from mistakes in a no-risk environment. By practicing in simulated settings, providers can identify and correct errors before encountering real patients. This practice enhances individual competency and promotes a safety culture within healthcare institutions. A review by Owen^[2]^ highlighted that patients generally support SBT because they understand its potential to reduce medical errors and improve patient outcomes. The realism of simulations is another critical factor influencing patient perspectives. Patients particularly appreciate high-fidelity simulations that closely mimic real-life medical scenarios, as they believe these provide the most effective training. However, there are concerns about the limitations of simulations in replicating the full complexity of human physiology and the unpredictability of clinical environments. Patients may worry that while simulations are beneficial, they might need to prepare providers for the nuances of actual patient care fully.^[3]^ while high-fidelity simulations are advancing, there is still a gap between simulated and real-world experiences, affecting patient perceptions of their efficacy. Ethical considerations in SBT also play a role in shaping patient perspectives. Patients expect that simulation training adheres to high ethical standards, particularly concerning the treatment of standardized patients and the confidentiality of simulation performance data. Standardized patients, who are actors trained to portray real patient cases, must be treated respectfully, and their participation should be ethical. Additionally, patients expect the data generated from simulation exercises to be used constructively to improve training programs without compromising privacy.^[4]^ discussed the importance of informed consent and ethical treatment in simulation training, noting that ethical transparency helps build patient trust. Cost is another consideration from the patient’s perspective. The advanced technology and resources required for effective SBT can be expensive, raising concerns about the potential for increased healthcare costs. Patients might worry that these costs will be passed on to them. Healthcare providers and educators must communicate the long-term benefits of SBT, such as reduced medical errors and improved clinical outcomes, to justify these costs. According to,^[5]^ when patients understand the cost-effectiveness of SBT in the long run, they are more likely to support its implementation despite the initial financial implications. Cultural differences also influence patient perspectives on SBT. In some cultures, there may be a preference for experiential learning through direct patient care rather than simulated practice. This preference can lead to skepticism about the effectiveness of SBT. Understanding and addressing these cultural nuances is essential for the broader acceptance of simulation training across different patient populations.^[6]^ highlighted the need for culturally sensitive approaches in designing and implementing SBT programs to ensure they are well-received by diverse patient groups. Engaging patients in designing and evaluating SBT programs can enhance their acceptance and relevance. When patients are involved in providing feedback and shaping the training scenarios, they feel a sense of partnership with healthcare providers. This engagement improves the quality of training by aligning it with real patient needs and fosters greater trust and satisfaction. Research by Scalese et al^[7]^ suggests that patient involvement in SBT can lead to more patient-centered training programs, ultimately benefiting learners and patients.

## 11. Interprofessional collaboration in SBT

Interprofessional collaboration in SBT is essential for preparing healthcare professionals to work effectively in multidisciplinary teams, improving patient outcomes and safety. This collaborative approach involves professionals from various healthcare disciplines, such as physicians, nurses, pharmacists, and allied health professionals, working to address patient care needs in simulated environments. Interprofessional SBT focuses on developing communication, teamwork, and leadership skills among participants, ultimately enhancing the quality and coordination of patient care. One of the key benefits of interprofessional SBT is improved communication among healthcare team members. Effective communication ensures seamless care coordination, prevents medical errors, and promotes patient safety. In simulated scenarios, healthcare professionals can practice communication skills, such as active listening, clear and concise verbal communication, and effective information sharing. Research by Bradley^[1]^ has shown that interprofessional SBT leads to better communication and collaboration among team members, which improves patient outcomes.

Teamwork is another essential aspect of interprofessional SBT. In healthcare, teams often comprise individuals from different disciplines who must work together cohesively to provide comprehensive care. Simulated scenarios allow healthcare professionals to practice teamwork skills in a controlled environment, such as task delegation, mutual respect, and conflict resolution. Team members learn to leverage each other’s strengths and expertise by working together in simulations, leading to more efficient and effective patient care. Studies by Owen^[2]^ have demonstrated that interprofessional SBT improves teamwork attitudes and behaviors among healthcare professionals, enhancing patient satisfaction and safety. Leadership development is also a focus of interprofessional SBT. Effective leadership is essential for guiding healthcare teams, making critical decisions, and ensuring patient-centered care. Simulated scenarios allow participants to assume leadership roles, delegate tasks, and prioritize patient care needs. Through feedback and debriefing sessions, participants can reflect on their leadership skills and identify areas for improvement. Research by Higham^[3]^ has shown that interprofessional SBT enhances leadership competencies among healthcare professionals, leading to more confident and effective leaders in clinical practice. Interprofessional SBT promotes a culture of mutual respect and understanding among healthcare professionals. Participants gain insight into other team members’ roles, responsibilities, and perspectives by working together in simulated scenarios. This increased awareness fosters a collaborative mindset and encourages interdisciplinary collaboration in real-world clinical settings. Moreover, interprofessional SBT promotes teamwork and cooperation, essential for addressing complex healthcare challenges and improving patient outcomes. Studies by Cooper and Taqueti^[4]^ have shown that interprofessional SBT enhances mutual respect and trust among healthcare professionals, leading to more effective interdisciplinary teamwork.

## 12. Continuing education and professional development through simulation

Continuing education and professional development through Simulation is vital to lifelong learning for healthcare professionals. SBT offers a dynamic and interactive approach to continuing education, allowing practitioners to refine existing skills, learn new techniques, and stay abreast of advances in medical practice. Through simulated scenarios, healthcare professionals can engage in realistic and immersive learning experiences replicating clinical situations, providing opportunities for deliberate practice, reflection, and skill enhancement. One of the primary benefits of continuing education through Simulation is the opportunity for hands-on practice in a safe and controlled environment. Healthcare professionals can engage in simulated scenarios that mirror real-world clinical situations, allowing them to apply their knowledge and skills in a risk-free setting. This hands-on experience enables practitioners to gain confidence, improve clinical competency, and enhance patient care outcomes. Research by Bradley^[1]^ has shown that simulation-based continuing education significantly improves clinical performance and patient safety. Additionally, simulation-based continuing education provides opportunities for interprofessional learning and collaboration. Healthcare professionals from different disciplines can participate in simulated scenarios, fostering teamwork, communication, and mutual respect. Interprofessional collaboration is essential for delivering high-quality patient care, and Simulation offers a platform for practitioners to develop and refine their collaborative skills in a supportive environment. Studies by Owen^[2]^ have demonstrated that interprofessional simulation-based education enhances teamwork attitudes and behaviors among healthcare professionals, improving patient outcomes. Another advantage of continuing education through Simulation is the ability to tailor learning experiences to individual needs and learning styles. Simulated scenarios can be customized to address specific learning objectives, allowing practitioners to focus on areas needing additional support or practice. Moreover, simulation-based learning can accommodate various modalities, including visual, auditory, and kinesthetic learning, ensuring that educational content is accessible and engaging for all learners.^[3]^ emphasize the importance of personalized learning experiences in health professions education, noting that Simulation allows individualized feedback and remediation. Simulation-based continuing education also promotes a culture of lifelong learning and professional development among healthcare professionals. By engaging in ongoing training and skill development, practitioners demonstrate a commitment to maintaining high standards of practice and providing the best possible care for their patients. Simulation offers a flexible and convenient approach to continuing education, allowing practitioners to access training opportunities conveniently. This accessibility encourages active participation in lifelong learning and supports career advancement within the healthcare profession.^[4]^ highlight the importance of judicious simulation technology in continuing medical education, noting its potential to enhance clinical skills and improve patient care outcomes. Furthermore, simulation-based continuing education contributes to the overall quality and safety of healthcare delivery. Practitioners participating in regular training and professional development are better equipped to identify and address clinical challenges, mitigate medical errors, and improve patient outcomes. By continuously refining their skills and staying updated with the latest evidence-based practices, healthcare professionals can provide more effective and efficient care, ultimately benefiting patient populations.^[5]^ emphasize the critical role of simulation-based medical education in improving clinical competencies and reducing medical errors.

## 13. Innovative uses of simulation technology

One innovative use of simulation technology is VR simulations, which provide healthcare professionals with immersive and interactive learning experiences. VR simulations replicate real-world clinical environments, allowing learners to practice clinical skills, procedures, and decision-making in a realistic and controlled setting. VR technology enables learners to interact with lifelike patient avatars, medical equipment, and diagnostic tools, enhancing the fidelity and authenticity of the learning experience. Research by Dhar et al^[8]^ has shown that VR simulations improve healthcare learners’ procedural skills, clinical judgment, and confidence. Another innovative application of simulation technology is gamification, which involves incorporating game elements and mechanics into educational simulations to enhance engagement, motivation, and learning outcomes. Gamified simulations often feature challenges, rewards, and feedback mechanisms encouraging active participation and skill development. By gamifying learning experiences, healthcare educators can create dynamic and interactive training environments that cater to modern learners’ preferences and learning styles. Studies by Owen^[2]^ and Al-Elq^[5]^ have demonstrated the effectiveness of gamified simulations in improving learning retention and performance. Telemedicine is another area where simulation technology is innovatively applied to enhance healthcare education and practice. Telemedicine simulations enable learners to remotely engage in patient encounters, consultations, and diagnostic assessments using telecommunication technologies. These simulations provide opportunities for learners to develop telemedicine skills, such as effective communication, remote diagnosis, and virtual patient management. Telemedicine simulations also support interdisciplinary collaboration and teamwork, as healthcare professionals from different locations can collaborate in real time to deliver patient care. Research by Cipresso et al^[9]^ has highlighted the benefits of telemedicine simulations in improving access to healthcare education and expanding clinical training opportunities. Simulation technology is also being used innovatively to address emerging challenges in healthcare delivery, such as the COVID-19 pandemic. Simulated scenarios allow healthcare professionals to practice and refine their responses to public health crises, infectious disease outbreaks, and disaster situations in a safe and controlled environment. These simulations help prepare healthcare teams for real-world emergencies, ensuring they are equipped to provide timely and effective patient care while minimizing risks to themselves and others. Research by Zackoff et al^[10]^ has emphasized the importance of SBT in pandemic preparedness and response efforts.

Furthermore, simulation technology is harnessed to support ongoing professional development and lifelong learning for healthcare professionals. Virtual simulation platforms offer convenient and accessible training opportunities for practitioners to engage in self-directed learning, skill assessment, and competency maintenance. These platforms enable practitioners to access high-quality educational content, participate in interactive learning modules, and receive personalized feedback and coaching. By leveraging simulation technology for professional development, healthcare professionals can stay updated with the latest evidence-based practices, expand their clinical competencies, and enhance patient care outcomes. Research by Al-Elq^[5]^ has highlighted the potential of virtual simulation platforms in supporting continuous learning and performance improvement.

## 14. Faculty development and support for SBT

One key aspect of faculty development for SBT is training in simulation pedagogy and instructional design. Educators need to understand the principles of adult learning theory, simulation-based teaching strategies, and best practices for designing compelling simulation scenarios. Training programs should cover scenario development, debriefing techniques, learner assessment, and simulation technology integration. By equipping faculty with pedagogical expertise, institutions can ensure that simulation-based education aligns with educational objectives, promotes active learning, and enhances learner engagement. Research by Bradley^[1]^ has emphasized the importance of faculty training in simulation pedagogy to improve simulation-based education quality. Another essential aspect of faculty development for SBT is technical training and proficiency in simulation technology. Educators need to be proficient in operating simulation equipment, managing simulation environments, and troubleshooting technical issues that may arise during simulation sessions. Training programs should provide hands-on experience with simulation technology, simulation software platforms, and audiovisual equipment.

Additionally, faculty members should receive training on using simulation-based assessment tools and data collection methods to evaluate learner performance and track educational outcomes. Research by Owen^[2]^ has highlighted the importance of technical training for faculty members involved in simulation-based education. Debriefing skills are also critical for faculty members engaged in SBT. Debriefing is a structured reflective process that occurs after simulation sessions, allowing learners to review their performance, identify areas for improvement, and engage in self-directed learning. Faculty members need to be trained in debriefing techniques, such as advocacy inquiry and debriefing with good judgment, to facilitate productive discussions and promote deep learning among learners. Training programs should provide opportunities for faculty members to practice debriefing skills through simulated scenarios and receive feedback from experienced debriefers. Research by Higham^[3]^ has highlighted the importance of debriefing training for faculty development in simulation-based education. In addition to pedagogical and technical training, faculty development for SBT should include opportunities for ongoing mentorship, peer support, and professional networking. Mentoring programs pair experienced simulation educators with novice faculty members to provide guidance, feedback, and support as they develop their skills and confidence in simulation-based teaching. Peer support networks, such as simulation interest groups and communities of practice, offer opportunities for faculty members to share resources, exchange ideas, and collaborate on educational initiatives. Professional networking events, conferences, and workshops provide platforms for faculty members to learn from experts in the field, stay updated with emerging trends in simulation education, and engage in scholarly activities. Research by Cooper and Taqueti^[4]^ has underscored the importance of mentorship and peer support in faculty development for SBT.

Furthermore, faculty development programs should address diversity, equity, and inclusion issues in simulation-based education. Educators must be aware of cultural, social, and linguistic factors that may impact learner experiences and outcomes in simulation scenarios. Training programs should emphasize the importance of creating inclusive learning environments, respecting learners’ backgrounds and identities, and addressing unconscious bias in simulation-based education. Faculty members should receive training on cultural competency, sensitivity to diversity, and strategies for promoting inclusivity in simulation sessions. Research by Al-Elq^[5]^ has highlighted the need for faculty development programs to address diversity and cultural competence issues in simulation-based education.

## 15. Student perspectives on SBT

One of the key benefits of SBT from the student perspective is the opportunity for hands-on, experiential learning in a safe and controlled environment. Simulation scenarios allow students to apply theoretical knowledge to practical, real-life situations, fostering the development of clinical skills, critical thinking, and decision-making abilities. By engaging in simulated patient encounters, procedures, and emergencies, students gain confidence, competence, and preparedness for clinical practice. Research by Datta et al^[6]^ has highlighted the positive impact of Simulation on student learning outcomes and clinical performance. Moreover, students appreciate the immersive and interactive nature of simulation-based learning, which promotes active engagement and participation. Simulation scenarios are designed to be realistic and engaging, capturing students’ attention and motivating them to invest fully in the learning process. Through Simulation, students can experience the complexities and challenges of healthcare delivery firsthand, preparing them to navigate similar situations in clinical practice. Studies by Scalese et al^[7]^ have demonstrated that Simulation enhances student engagement, motivation, and satisfaction with learning.

Additionally, students value the opportunity for repeated practice and feedback provided by simulation-based education. Unlike traditional clinical experiences, where practice opportunities may be limited, Simulation allows students to repeat scenarios, refine their skills, and learn from their mistakes in a supportive environment. Feedback from faculty and peers during debriefing sessions enables students to reflect on their performance, identify improvement areas, and set future learning goals. This iterative process of practice and feedback fosters deep learning and skill mastery among students. Research by Dhar et al^[8]^ has emphasized the importance of feedback in simulation-based education for promoting skill acquisition and performance improvement.

Furthermore, students appreciate the interprofessional learning opportunities facilitated by simulation-based education. Interprofessional collaboration is essential for providing comprehensive, patient-centered care, and Simulation offers a platform for students from different disciplines to work together as healthcare team members. Through simulated scenarios, students learn to communicate effectively, collaborate with colleagues, and understand the roles and responsibilities of various healthcare professionals. Interprofessional simulation experiences promote mutual respect, interdisciplinary understanding, and teamwork skills essential for successful clinical practice. Studies by Cipresso et al^[9]^ have highlighted the benefits of interprofessional Simulation for enhancing student collaboration and teamwork attitudes. Despite the many benefits of SBT, students also acknowledge certain challenges and limitations associated with this educational approach. One common concern is the fidelity of simulation scenarios compared to real-life clinical encounters. While simulations strive to replicate clinical situations, they may need to fully capture the complexity, unpredictability, and emotional intensity of actual patient care experiences. Students may worry that simulation-based learning needs to prepare them for the realities of clinical practice adequately. Research by Zackoff et al^[10]^ has discussed the limitations of simulation fidelity and its implications for student learning and clinical preparedness. Another challenge students cite is the cost and resource-intensive nature of simulation-based education. Developing and maintaining simulation facilities, purchasing equipment and supplies, and training faculty require significant financial investment from educational institutions. Students may question the cost-effectiveness of simulation programs and express concerns about the equitable distribution of resources across academic programs. Educators and administrators need to justify the costs of simulation-based education by demonstrating its impact on student learning outcomes, clinical competency, and patient care quality. Studies by Chang et al^[11]^ have addressed the financial implications of simulation-based education and strategies for maximizing return on investment.

## 16. Future directions and innovations

AI is poised to revolutionize SBT by offering opportunities for personalized learning experiences and adaptive training programs. AI-driven simulations have the potential to provide real-time feedback, monitor learners’ progress, and tailor training scenarios to individual skill levels and learning needs. By leveraging machine learning algorithms, AI can analyze learners’ performance data and identify areas for improvement, allowing for more targeted and efficient training interventions.^[1]^ Moreover, AI can enhance the realism and responsiveness of simulation scenarios by simulating dynamic patient responses and adapting scenarios based on learners’ actions, creating a more immersive and engaging learning experience.^[2]^ Advancements in VR and AR applications are also shaping the future of SBT. VR and AR technologies offer unprecedented opportunities to create highly realistic and immersive simulation environments resembling clinical settings. VR simulations allow learners to interact with virtual patients, medical equipment, and clinical environments in a safe and controlled manner, providing hands-on experience without physical resources.^[3]^ AR applications, on the other hand, overlay digital information onto the real-world environment, enhancing learners’ situational awareness and decision-making skills during simulated scenarios.^[4]^ These technologies are increasingly integrated into simulation training programs to improve realism, engagement, and learning outcomes. The integration of Simulation with other training modalities represents another essential direction for the future of medical education. Blended learning approaches that combine Simulation with traditional classroom instruction, hands-on clinical experience, and online learning modules offer a comprehensive and flexible approach to training. Blended learning allows learners to benefit from the strengths of each modality, leveraging Simulation for experiential learning, traditional lectures for theoretical knowledge acquisition, and clinical rotations for practical application and reinforcement.^[5]^ Interdisciplinary training programs that bring together learners from different healthcare professions, such as medicine, nursing, and allied health, are also gaining traction. These programs promote collaboration, communication, and teamwork among healthcare professionals, reflecting the interdisciplinary nature of modern healthcare delivery.^[6]^ By integrating Simulation into multidisciplinary training programs, educators can prepare learners for the dynamic and complex challenges they encounter in interprofessional practice settings. Global perspectives are increasingly shaping the future of SBT, with efforts focused on expanding access to simulation training in developing countries and adapting simulation programs to diverse cultural contexts. In many low- and middle-income countries, access to high-quality medical education and training resources is limited, posing significant challenges for healthcare workforce development. SBT offers a scalable and cost-effective solution to address these challenges, providing learners hands-on experience and skills development in a safe and controlled environment.^[7]^ Initiatives such as the World Health Organization’s Global Initiative for Emergency and Essential Surgical Care promote SBT in resource-limited settings, improving surgical and anesthesia care.^[8]^ Additionally, efforts are underway to adapt simulation programs to diverse cultural contexts, considering linguistic, socio-cultural, and healthcare system differences. Cultural competency training using Simulation can help learners develop the skills and attitudes needed to provide culturally sensitive, patient-centered care.^[9]^

## 17. Case studies and research findings

One notable case study is from the University of Miami’s Gordon Center for Research in Medical Education. This institution implemented a comprehensive SBT program for emergency medicine residents. The program included high-fidelity simulations of various emergency scenarios such as cardiac arrest, trauma, and airway management. The results were impressive: residents who participated in the simulation training demonstrated significantly improved performance in real-life emergencies. They were quicker to recognize and respond to critical conditions and made fewer errors than their peers who underwent traditional training.^[1]^ Another success story comes from the Mayo Clinic, which integrated SBT into its anesthesiology residency program. Using high-fidelity mannequins, residents practiced managing complex anesthesia-related emergencies, including malignant hyperthermia and difficult airway scenarios. The training led to a marked improvement in residents’ confidence and competence. Subsequent evaluations showed that these residents were better prepared to handle actual clinical emergencies, which enhanced patient safety and care quality.^[2]^ A third case study is simulation training at the Veterans Health Administration. The Veterans Health Administration implemented a standardized patient program to train healthcare providers in communication skills, particularly for end-of-life care discussions. The program utilized actors trained to portray patients and family members in emotionally charged scenarios. The feedback from participants indicated significant improvements in their communication skills, empathy, and ability to handle difficult conversations. The program improved the quality of patient-provider interactions and contributed to higher patient satisfaction rates.^[3]^ These success stories underscore the transformative impact of SBT in medical education. They demonstrate how Simulation can enhance technical and non-technical skills, leading to better preparedness and improved clinical outcomes. However, these case studies also offer valuable lessons. One key lesson is the importance of integrating Simulation into the broader curriculum. Successful programs are those where Simulation is not an isolated activity but is woven into the fabric of the educational experience, reinforcing learning objectives and complementing other training methods.^[4]^ Empirical research further supports the effectiveness of SBT. A comprehensive meta-analysis by Cook et al reviewed over 600 studies comparing SBT to traditional methods. The findings were unequivocal: simulation training was associated with improved knowledge, skills, and behaviors across various clinical disciplines. Notably, the benefits were most pronounced when Simulation was combined with other instructional methods and when feedback and deliberate practice were incorporated.^[40]^ Comparative studies have also highlighted the advantages of Simulation over traditional training methods. For example, a study by Wayne et al compared the performance of internal medicine residents trained in advanced cardiac life support (ACLS) using Simulation versus traditional lecture-based instruction. The simulation-trained group performed significantly better in simulated and real-life cardiac arrest scenarios. They adhered more closely to ACLS guidelines, initiated critical interventions more promptly, and demonstrated superior team coordination.^[41]^ Another comparative study focused on surgical training. Seymour et al evaluated the impact of VR simulation on laparoscopic surgery skills. Residents trained using VR simulators performed markedly better in the operating room than those who received standard training. The VR-trained residents completed procedures faster, made fewer errors, and exhibited greater precision. This study highlighted the potential of VR simulation to bridge the gap between theoretical knowledge and practical skills in a controlled, reproducible environment.^[42]^ SBT has also enhanced non-technical skills crucial for effective healthcare delivery. For instance, a study by Fernandez et al examined the impact of team-based simulation training on communication and teamwork in emergency department teams. The results indicated significant improvements in team communication, coordination, and overall performance during high-stress, high-stakes situations. This is particularly important given the growing recognition that non-technical skills are critical determinants of patient safety and quality of care.^[43]^ Despite the compelling evidence supporting SBT, it is important to acknowledge some of its limitations. One challenge is the potential variability in training quality. The effectiveness of Simulation can vary depending on the simulator’s fidelity, the scenarios’ realism, and the instructors’ expertise. Ensuring consistent, high-quality simulation experiences requires substantial investment in training faculty and maintaining equipment.^[9]^ Furthermore, while Simulation can replicate many aspects of clinical practice, it cannot fully capture the complexity and unpredictability of real-life patient care. This limitation highlights the importance of using Simulation as a complement to, rather than a replacement for, traditional clinical training. Combining Simulation with hands-on clinical experience gives learners a more comprehensive educational experience.^[10,44]^

## 18. Conclusion and call to action

SBT in medical education represents a transformative approach to teaching and learning, leveraging advanced technologies and innovative methodologies to enhance the competence and confidence of healthcare professionals. As detailed, integrating high-fidelity simulators, VR, AR, standardized patients, and hybrid simulations offers a multifaceted framework for developing technical and non-technical skills essential for patient care. The benefits of these modalities are significant, ranging from improved clinical outcomes and increased patient safety to enhanced communication and teamwork abilities. However, the successful implementation of SBT has its challenges. High costs, the need for specialized equipment and facilities, faculty training, and the limitations in realism compared to actual clinical settings are critical barriers that must be addressed. By acknowledging and strategically managing these challenges, educational institutions can maximize the potential of SBT to prepare future healthcare professionals more effectively. Technological advancements such as AI, improved VR and AR applications, and innovative blended learning approaches hold promise for the future, enabling more personalized, adaptive, and immersive learning experiences. Furthermore, expanding SBT’s reach to developing countries and adapting programs to diverse cultural contexts are essential steps toward achieving global equity in healthcare education.

## 19. Call to action

To fully realize the potential of SBT in medical education, stakeholders across the healthcare and educational sectors must take concerted action. Here are several key recommendations:

*Investment in technology and infrastructure*: Governments, educational institutions, and private sectors should invest in technology and infrastructure to support high-fidelity simulators, VR, and AR applications. Funding should also be allocated for the maintenance and upgrading of these systems to ensure long-term sustainability.

*Faculty development and training*: Comprehensive training programs for instructors are essential. Institutions should prioritize the development of faculty expertise in simulation technologies and pedagogical techniques to enhance the quality and effectiveness of SBT.

*Curriculum integration*: Simulation should be seamlessly integrated into medical curricula, complementing traditional teaching methods and clinical experiences. Interdisciplinary training programs that include multiple healthcare professions can foster teamwork and collaboration, reflecting real-world clinical environments.

*Research and evaluation*: Continuous research and evaluation are critical to assess the effectiveness of SBT and identify areas for improvement. Rigorous studies should be conducted to measure the impact of Simulation on clinical performance and patient outcomes, and findings should be used to refine training programs.

*Global collaboration and access*: Efforts should be made to expand access to SBT in low- and middle-income countries. International collaborations, funding initiatives, and culturally adapted programs can help bridge the gap in healthcare education and improve global health outcomes.

*Public and private sector partnerships*: Establishing partnerships between academic institutions, healthcare providers, technology companies, and policymakers can facilitate resource sharing, innovation, and the development of best practices in SBT.

## Acknowledgments

The authors would like to express gratitude to all individuals and institutions that contributed to the completion of this paper. Their support, guidance, and encouragement throughout the research process are deeply appreciated.

## Author contributions

**Conceptualization:** Chukwuka Elendu, Dependable C. Amaechi, Emmanuel C. Amaechi, Tochi C. Elendu, Ijeoma D. Elendu.

**Data curation:** Chukwuka Elendu, Dependable C. Amaechi, Emmanuel C. Amaechi, Tochi C. Elendu, Ijeoma D. Elendu.

**Formal analysis:** Chukwuka Elendu, Dependable C. Amaechi, Emmanuel C. Amaechi, Tochi C. Elendu, Ijeoma D. Elendu.

**Funding acquisition:** Chukwuka Elendu, Dependable C. Amaechi, Emmanuel C. Amaechi, Tochi C. Elendu, Ijeoma D. Elendu.

**Investigation:** Chukwuka Elendu, Dependable C. Amaechi, Emmanuel C. Amaechi, Tochi C. Elendu, Ijeoma D. Elendu.

**Methodology:** Chukwuka Elendu, Dependable C. Amaechi, Emmanuel C. Amaechi, Tochi C. Elendu, Ijeoma D. Elendu.

**Project administration:** Chukwuka Elendu, Dependable C. Amaechi, Emmanuel C. Amaechi, Tochi C. Elendu, Ijeoma D. Elendu.

**Resources:** Chukwuka Elendu, Dependable C. Amaechi, Emmanuel C. Amaechi, Tochi C. Elendu, Ijeoma D. Elendu.

**Software:** Chukwuka Elendu, Dependable C. Amaechi, Emmanuel C. Amaechi, Tochi C. Elendu, Ijeoma D. Elendu.

**Supervision:** Chukwuka Elendu, Dependable C. Amaechi, Alexander U. Okatta, Emmanuel C. Amaechi, Tochi C. Elendu, Chiamaka P. Ezeh, Ijeoma D. Elendu.

**Validation:** Chukwuka Elendu, Dependable C. Amaechi, Alexander U. Okatta, Emmanuel C. Amaechi, Tochi C. Elendu, Chiamaka P. Ezeh, Ijeoma D. Elendu.

**Visualization:** Chukwuka Elendu, Dependable C. Amaechi, Alexander U. Okatta, Emmanuel C. Amaechi, Tochi C. Elendu, Chiamaka P. Ezeh, Ijeoma D. Elendu.

**Writing – original draft:** Chukwuka Elendu, Dependable C. Amaechi, Alexander U. Okatta, Emmanuel C. Amaechi, Tochi C. Elendu, Chiamaka P. Ezeh, Ijeoma D. Elendu.

**Writing – review & editing:** Chukwuka Elendu, Dependable C. Amaechi, Alexander U. Okatta, Emmanuel C. Amaechi, Tochi C. Elendu, Chiamaka P. Ezeh, Ijeoma D. Elendu.
